# Breastfeeding and complementary feeding practices in Madagascar: results of a secondary analysis of the freeBily trial

**DOI:** 10.1186/s13006-026-00861-6

**Published:** 2026-06-12

**Authors:** Valentina Marchese, André Brito, Raphäel Rakotozandrindrainy, Norbert Georg Schwarz, Njary Rakotozandrindrainy, Tahinamandranto Rasamoelina, Mala Rakoto Andrianarivelo, Jeannine Solonirina, Elveric Fesia Ratiaharison, Mickael Radomanana, Tiana Randrianarisoa, Philipp Klein, Jule Hameister, Leonard Gunga, Irina Kislaya, Anna Jaeger, Govert van Dam, Jürgen May, Rivo Andry Rakotoarivelo, Daniela Fusco, the freeBILy consortium

**Affiliations:** 1 Research Group: Implementation Research, Bernhard Nocht Institute for Tropical Medicine, Hamburg, Germany; 2 German Center for Infection Research (DZIF), Hamburg-Borstel-Lübeck-Riems, Hamburg, Germany; 3 University of Antananarivo, Antananarivo, Madagascar; 4 Department of Infectious Diseases Epidemiology, Bernhard Nocht Institute for Tropical Medicine (BNITM), Hamburg, Germany; 5 Centre d’Infectiolologie Charles Mérieux (CICM), Antananarivo, Madagascar; 6 Infectious Diseases Service, University Hospital Tambohobe, Fianarantsoa, Madagascar; 7 Leiden University Center for Infectious Diseases, Leiden University Medical Center, Leiden, The Netherlands

**Keywords:** Breastfeeding, Complementary feeding, Nutritional global targets, Maternal health, Child health, Madagascar, Resource-limited countries

## Abstract

Breastfeeding and complementary feeding are key public health practices for child development and attainment of optimal nutritional status, with additional beneficial effects on maternal health. In Madagascar, we conducted a secondary analysis of a cluster randomised clinical trial data (Pan-African Clinical Trial Register PACTR201905784271304) to assess the practice of delayed complementary feeding (DCF) at nine months postpartum (T3 visit) and continued breastfeeding (CB) at 24 months (T4). Factors associated with the prevalence of DCF were investigated using a generalized estimating equation (GEE) for binomial outcome with logarithmic link function to account for clustering, prevalence ratio (PR) with 95% confidence intervals (CIs) were estimated. A Cox proportional hazards model was used to assess factors associated with breastfeeding termination (BT). A total of 1863 women were evaluated at T3, 2332 at T4. At T3, 1848 (98.8%) were breastfeeding and 479 (25.7%) were delaying complementary feeding. At T4, 1269 (54.4%) were breastfeeding. Median time for BT was 21 months (range 2–25). Secondary education (PR = 0.67, CI 0.49–0.93) and anaemia (PR = 0.75, CI 0.65–0.88) were associated with lower prevalence of DCF at T3, being a farmer with a higher prevalence (PR = 3.45, CI 1.90–6.28). Older age was associated with longer CB (hazard ratio 0.68, CI 0.49–0.96 for 30–50 years). The association between being farmer mothers and DCF at nine months requires further investigations. Two main lines of public health actions are identified: health education, with intergenerational mentoring initiatives from older to younger women, and integrating maternal and child health programmes to improve both maternal anaemia and child development in a life-cycle approach.

**Supplementary information:**

The online version contains supplementary material available at 10.1186/s13006-026-00861-6.

## Introduction

Breastfeeding is an intrinsic part of the human experience and an essential public health practice to support child development, provide nutrition, to improve both child and maternal health [1] and to ensure adequate nutrition starting early in life [2]. The World Health Organization (WHO) recommends early breastfeeding within one hour after birth, exclusive breastfeeding until six months of age and continued breastfeeding coupled with an incremental introduction of complementary food up to two years and/or over [3]. Global targets have been developed, particularly for exclusive breastfeeding up to six months, which should be practiced worldwide for 50% of infants by 2025 [4]. These recommendations are based on growing evidence of the short- and long-term benefits of optimal breastfeeding practices [5]. Benefits range from a reduced incidence of respiratory infections and diarrheal episodes to the prevention of obesity and improved cognitive development. The benefits also extend to the health of the mother, for whom there is evidence of a reduced incidence of breast cancer, type II diabetes and hypertension [5].

Complementary feeding with nutrient-dense, safe, and diverse foods of animal and vegetal origin (not exclusively milk substitutes) should be introduced at six months while continuing breastfeeding [6]. The introduction of complementary foods after six months has been associated to low height and low body mass index (BMI), suggesting an association with increased risk of hospitalization or episodes of diarrhoea [6, 7].

Despite this evidence, breastfeeding and infant feeding practices still vary widely around the world. There are important differences between high income countries (HICs) and low and middle income countries (LMICs), which overall present longer breastfeeding duration [1], but a downward trend in the practice of introducing complementary food at six to eight months since the 2000s [5]. In both contexts, socio-economic, cultural and individual factors were associated with the adoption of appropriate breastfeeding practices [8–11]. The latest estimates for 2023 show a 10% increase over the previous decade, with an encouraging 48% achievement of exclusive breastfeeding at six months, close to the World Health Assembly target of 50% for the same year [12]. However, reaching the 2030 targets of 70% of children breastfed within the first hour of life and exclusively until six months, 80% until one year and 60% between 20 and 23 months is still a long way off [12].

Madagascar is one of the poorest countries in the world, with the highest levels of food insecurity among countries without conflict [13]. According to the latest Demographic and Health Survey (DHS), 40% of children suffer from chronic malnutrition and growth retardation, with the latter peaking in the 18–23-month age group [14]. In a context of such food insecurity and poverty, it is crucial to implement all possible measures to improve the nutritional and growth status of children. This includes the optimisation of breastfeeding and feeding practices under the age of two, age group also affected by the highest level of growth retardation according to DHS. Awareness initiatives and legal protections already exist for breastfeeding promotion and support. This includes the right to 14 weeks of maternity leave and one hour of breastfeeding per day for the first 15 months of a child’s life for working mothers [15, 16]. Nevertheless, political commitment to support breastfeeding needs to be strengthened, both through adequate financial support and through appropriate campaigns to raise awareness and understanding of the factors related to good breastfeeding practices in the country [17].

This study describes breastfeeding practices and investigates the factors associated with delayed complementary feeding and breastfeeding termination. The main goal of the analysis is to inform stakeholders to develop public health interventions aimed at tailoring breastfeeding recommendations in the country to ultimately improve mother and child health.

## Materials and methods

### Study design and population

We performed secondary data analysis within a phase III, two-arm, cluster randomised clinical trial (CRCT), “fast and reliable easy-to-use diagnostics for eliminating bilharzia in young children and mothers” (freeBILy). Details on the freeBILy design and methods has been published elsewhere [18].

Briefly, pregnant women were recruited in three regions: Itasy and Bongolava (west of Antananarivo), and Amoron’i Mania (north of Fianarantsoa). The study was implemented during antenatal care at 42 primary health care centers (PHCCs) (40 randomized and 2 pilot sites) and included five visits: T0 (recruitment), T1 (in the 8th month of pregnancy), T2 (at delivery when the child/children were also enrolled), T3 (nine months after delivery), and T4 (24 months after delivery) [18]. Inclusion criteria for recruitment were: women being at least 16 years old, between 5^th^ and 6^th^ months of gestation, able to give written informed consent, expected to stay in the study area for the next 24 months, born to an enrolled mother (for children). Exclusion criteria were: not being pregnant or having a gestational age out of the specified range, a history of blood transfusion, congenital anaemia, or seizures for mother, and being born in a PHCC other than the one in which the mother was enrolled (for children). We additionally excluded women with a history of a new pregnancy/birth during the follow-up period and those in whom the child/children were out of 9 (range 8–10) month and 24 (range 23–26) month age interval at the time of the T3 and T4 follow-up visits, respectively.

### Data collection and outcomes

Trained health professionals at the PHCCs used paper-based case report forms to collect women’s sociodemographic information, data on health status, past and current medical history, and blood samples for haemoglobin evaluation. Data collection took place in April 2020-April 2021 for T3 and in June 2021-August 2022 for T4. The data were digitised in REDCap [19] on the basis of a double data entry procedure and were checked for completeness and consistency by a data quality manager.

The women were asked if they were breastfeeding at the time of T3 and T4 visits and if the breastfeeding was exclusive, i.e., no complementary food was introduced. If breastfeeding was interrupted, the month of interruption was recorded. The responses were used to assess the following outcomes: continued breastfeeding (CB), defined as ongoing breastfeeding at 24 months (with or without any complementary feeding); delayed complementary feeding (DCF) at T3 (nine months), described as breastfeeding without any other complementary feeding, and breastfeeding termination (BT), expressed as month of age of the child when the cessation of breastfeeding occurred. Women were also asked if they were taking iron or folic acid supplements and their haemoglobin values were assessed by means of the POC Hemocue in both follow-up visits [20]. Anaemia status was defined for haemoglobin concentration < 120 g/L and classified according to WHO recommendations as follows: mild (110–119 g/L), moderate (80–109 g/L) and severe (<80 g/L) [21].

For children’s health, mothers were asked if they had any episodes of diarrhoea and, if so, how many, and if they had to be hospitalised, children weight and length were measured.

### Statistical analysis

The characteristics of the participants were summarised using frequencies and percentages for categorical variables, and measures of central tendency and dispersion for numerical variables. Prevalence of DCF and CB were estimated with respective 95% confidence intervals (CIs) stratified by maternal characteristics. Factors associated with the DCF were assessed at T3 using a GEE (generalized estimating equations) for a binomial family with logarithmic link function, to account for clustering. Crude (cPR) and adjusted prevalence ratios (aPR) with 95% CIs were estimated. Cumulative probability of breastfeeding was estimated using the Kaplan–Meier method at T4. To assess factors associated with breastfeeding termination at T4 a Cox proportional hazards model was used, hazard ratios (HR) with CIs were estimated. The proportional hazard assumption was checked using the Schoenfeld residuals test. As potential confounders, we considered urbanization, maternal age, education, occupation, and anaemia status and child sex in all multivariable models. These variables were identified as potentially relevant for breastfeeding practices based on the existing literature and included in the multivariable regression models regardless of the significance in bivariate analysis. Multicollinearity was assessed using VIF (variance inflation factors). Participants with missing values in any of the variables required for a given model were excluded from the analysis. Data analysis was performed using R software, version 4.4.0.

## Results

### Characteristics of the study sample

**Fig. 1:**
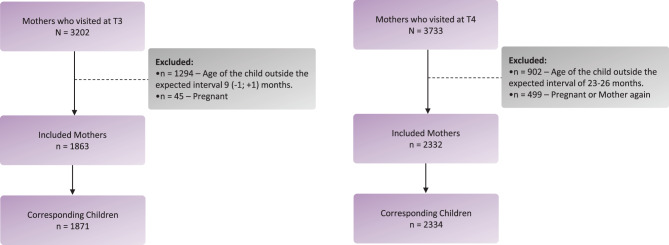
Participants flow-chart for the inclusion in the analysis

During the freeBILy trial 3402 women were evaluated at T3 and 3733 at T4. After assessing eligibility, data from 1863 mothers at T3 and 2332 at T4 were included in the analysis, corresponding to 1871 and 2334 children, respectively. A detailed flowchart for participants’ inclusion is reported in Figure 1 (Fig. 1).

At both visits, most mothers were from non-urban areas (74.8% at T3, 69.4% at T4), the great majority of cases had some kind of education (>48% primary and 47.6% secondary education at both visits), were employed at recruitment, (93.7% and 90.4%), and about 70% were farmers (71.7% and 68.4%) (Table 1). For about 30% of the mothers the child included in the study was their first child at both visits. Nearly 50% of the women were found to have some degree of anaemia, which was moderate or severe in at least 15% of cases at both visits (17.4% and 14.6%, respectively). The history of diarrhoea among the children varied from 34.1% to 53.9% between T3 and T4. However, the mean number of episodes was low (1.64 and 1.76 in T3 and T4, respectively), with hospitalisation percentages below 0.5% at both visits (Table 2).

**Table 1:** Socio-demographics and clinical characteristics of mothers at T3 (9±1 months) and T4 (23–26 months) after delivery

Characteristics	Visit T3 (9 months)-	Visit T4 (24 months)-
	Mothers (N = 1863)	Mothers (N = 2332)
**Region**		
Amoron’i Mania Region	809 (43.4%)	911 (39.1%)
Bongolava	179 (9.6%)	280 (12.0%)
Itasy	875 (47.0%)	1141 (48.9%)
**Urbanization**		
Non-urban	1393 (74.8%)	1619 (69.4%)
Urban	470 (25.2%)	713 (30.6%)
**Age Groups, years**		
(15,20)	257 (13.8%)	82 (3.6%)
(20,25)	634 (34.0%)	881 (37.8%)
(25,30)	481 (25.8%)	651 (27.9%)
(30,50)	491 (26.4%)	718 (30.7%)
**Education**		
Never went to school	68 (3.7%)	89 (3.8%)
Primary school	909 (48.8%)	1133 (48.6%)
Secondary school or higher	886 (47.6%)	1110 (47.6%)
**Employment**		
Unemployed	118 (6.3%)	223 (9.6%)
Employed (Non-Farmer)	410 (22.0%)	514 (22.0%)
Employed (Farmer)	1335 (71.7%)	1595 (68.4%)
First Child		
No	1287 (69.1%)	1578 (67.7%)
Yes	576 (30.9%)	754 (32.3%)
**Twin birth**		
No	1851 (99.4%)	2316 (99.3%)
Yes	12 (0.6%)	16 (0.7%)
Missing	0 (0%)	0 (0%)
**Supplements (iron and folic acid)**		
No	1665 (89.4%)	2324 (99.7%)
Yes	198 (10.6%)	8 (0.3%)
**Anaemia (Hb < 120 g/dl)**		
No	934 (50.1%)	1187 (50.9%)
Yes	801 (43.0%)	1131 (48.5%)
Missing	128 (6.9%)	14 (0.6%)
**Anaemia severity**		
Severe	3 (0.2%)	5 (0.2%)
Moderate	320 (17.2%)	331 (14.2%)
Mild	478 (25.7%)	795 (34.1%)
Non-Anaemia	934 (50.1%)	1187 (50.9%)
Missing	128 (6.9%)	14 (0.6%)

**Table 2:** Socio-demographics, clinical characteristics and breastfeeding termination of children at T3 (9±1 months) and T4 (23–26 months) after delivery

Characteristics	Visit T3 (9 months)-	Visit T4 (24 months)-
	Children (N = 1871)	Children(N = 2334)
**Age (months)**		
Mean (SD)	9.14 (0.439)	24.9 (0.698)
Median [Min, Max]	9.17 [8.02, 9.99]	24.7 [23.10, 26.00]
**Sex**		
Male	957 (51.1%)	1158 (49.6%)
Female	914 (48.9%)	1176 (50.4%)
**Suffered from diarrhoea**		
No	1233 (65.9%)	1098 (47.0%)
Yes	638 (34.1%)	1234 (52.9%)
Doesn’t know	0 (0.0%)	2 (0.1%)
**Diarrhoea episodes#**		
Mean (SD)	1.64 (0.889)	1.76 (0.921)
Median [Q1, Q3]	1.00 [1.00, 2.00]	2.00 [1.00, 2.00]
Missing	2 (0.1%)	1 (0.1%)
**Was hospitalised**		
No	1867 (99.8%)	2328 (99.7%)
Yes	4 (0.2%)	6 (0.3%)
**Weight, kg**		
Mean (SD)	7.89 (0.971)	10.59 (1.054)
Median [Q1, Q3]	7.83 [7.24, 8.50]	10.55 [10.00, 11.20]
**Length, cm**		
Mean (SD)	68.43(3.057)	80.84 (4.547)
Median [Q1, Q3]	68.5 [67.00, 70.00]	81.00 [79.40, 82.90]
**Breastfeeding practices**		
**Age (months) when breastfeeding was terminated**		
Mean (SD)	NA	21.2 (2.8)
Median [Min Max]	NA	22.0 [2.00, 25.0]

# The number of diarrhoea episodes was assessed only among those reporting diarrhoea

### Breastfeeding and feeding practices

Breastfeeding was practiced by 98.8% (CI 97.9 - 99.4) of the women at T3 (Table S1). DCF was reported by 25.7% (CI 11.8 - 47.1) of the mothers at nine months (Table S1). DCF practice was higher among young mothers (15–20) years old (31.0%) compared to older age groups (24.6%, 30–50 years old), in rural settings (28.3% vs. 19.2%), among those working as farmers (37.9%), and among non-anaemic women (30.98% vs. 24.12%). We observed an educational gradient in DCF, with the frequency of DCF declining with an increase in maternal education level from 37.9% among those with no formal education to 21.42% among those with secondary school or higher. Complementary feeding practices were similar between male and female babies.

At T4 CB was reported by 54.4% (CI 95% 44.5 - 63.9) (Table S2). CB was more frequent in rural settings compared to urban ones (57.7% vs. 46.7%) and increased with women’s age from 41.0% among 15–20 years old to 60.6% among 30–50 years old. At T4 we observed a decline in the practice of CB with an increase in maternal education from 62.9% among women who never went to school to 50.5% among ones with secondary or higher education.

**Fig. 2:**
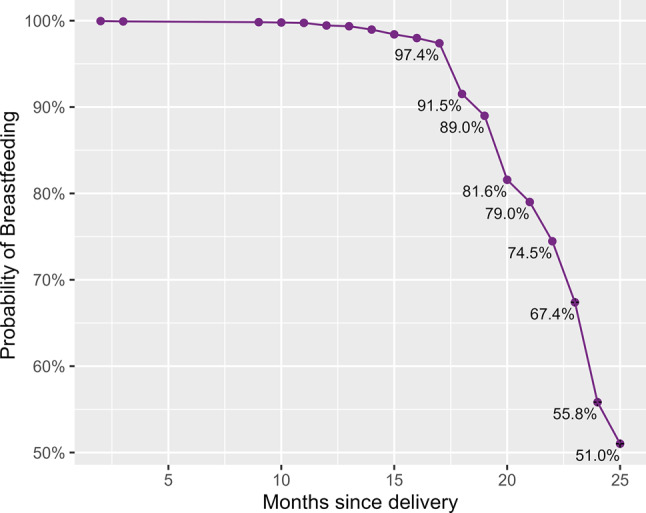
Cumulative breastfeeding probability by time since delivery (in months) estimated using the Kaplan–Meier (KM) method

Among the mothers who had interrupted breastfeeding before T4, the median time to BT was 22 months (range 2–25). Figure 2 shows the cumulative probabilities of breastfeeding up to 25 months after birth. The rates of breastfeeding remained high up to 17 months (97.4%, CI 96.7–98.0), while there was a rapid decline in breastfeeding from month 17 onwards (Fig. 2, Table S3).

### Factors associated to delayed complementary feeding

Table 3 shows crude and adjusted prevalence ratios of DCF at T3. After adjusting for confounding, we observed statistically significant associations of DCF with education, and health status. Having a secondary level of education and above (aPR = 0.67, CIs 0.49–0.93) and suffering from anaemia (aPR = 0.75, CIs 0.65–0.88) were associated with a lower prevalence of DCF at nine months, while being a farmer was positively associated with this practice (aPR = 3.45, CIs 1.90–6.28). Due to high frequency of breastfeeding at T3 (98.8%), cPR and aPR were not estimated for this outcome.

**Table 3:** Factors associated with prevalence of delayed complementary feeding at T3 visit (9±1 months)

	Delayed complementary feeding at T3 (*n* = 1716)					
Characteristics	cPR	CI	p	aPR	CI	p
**Urbanization**						
Rural	ref			ref		
Urban	0.68	0.54–0.85	**0.001**	0.95	0.76–1.18	0.643
**Education level**						
Never went to school	ref			ref		
Primary School	0.78	0.57–1.08	0.130	0.82	0.60–1.12	0.206
Secondary school or higher	0.55	0.40–0.77	**<0.001**	0.67	0.49–0.93	**0.017**
**Employment**						
Not employed	ref			ref		
Employed (Non-farmer)	1.36	0.71–2.62	0.357	1.41	0.74–2.69	0.294
Employed (Farmer)	3.59	1.95–6.60	**<0.001**	3.45	1.90–6.28	**<0.001**
**Age Group**						
(15,20)	ref			ref		
(20,25)	0.84	0.67–1.05	0.120	0.91	0.73–1.13	0.406
(25,30)	0.85	0.67–1.07	0.168	0.89	0.71–1.13	0.342
(30,50)	0.80	0.63–1.02	0.058	0.83	0.66–1.04	0.099
**Anaemia (Hb < 120)**						
No	ref			ref		
Yes	0.78	0.67–0.91	**0.002**	0.75	0.65–0.88	**<0.001**
**Child sex**						
Female	ref			ref		
Male	0.97	0.84–1.14	0.734	0.99	0.86–1.15	0.913

cPR – crude prevalence ratio, aPR - adjusted prevalence ratio, p- *p*-value, CI − 95% confidence interval

VIF: ranged from 1.01 to 1.09; Participants were excluded due to missing values in the following variables: anaemia, *n* = 128, delayed complementary feeding *n* = 27)

### Factors associated with breastfeeding termination at T4

Crude (cHR) and adjusted PR (aHR) of BT are presented in Table 3. In a bivariate analysis, the hazard of BT for mothers with secondary or higher education was 1.48 times greater for those who never went to school (cHR = 1.48, CI 1.03–2.12). In contrast, the hazard of breastfeeding termination was 34% lower for mothers aged 30–50 years old compared to those aged 15–20 years old (cHR = 0.66, CI 0.49–0.90). After adjustment for confounding, association of BT with secondary or higher level of education was attenuated (CIs1.00 -2.06), while being aged between 30 and 50 showed a strong evidence of association with BT in the Cox proportional hazards model, with an aHR of 0.68, CIs 0.49–0.96. This result suggests that mothers in the older age group had a lower probability of BT at the moment of the visit (Table 4) and were more likely to have an extended duration of breastfeeding.

**Table 4:** Multivariable Cox proportional hazard model analysis of time to the termination of breastfeeding at T4

Breastfeeding termination (n = 2320)						
Characteristics	Number of events	cHR	CI	aHR	CI	p
**Urbanization**						
Rural	679	ref		ref		
Urban	378	1.30	0.72–2.38	1.19	0.66–2.14	0.565
**Education leve**l						
Never went to school	32	ref		ref		
Primary School	479	1.20	0.84–1.73	1.23	0.86–1.77	0.259
Secondary school or higher	546	1.48	1.03–2.12	1.43	1.00–2.06	0.053
**Employment**						
Not employed	126	ref		ref		
Employed (Non-farmer)	275	1.13	0.86–1.48	1.17	0.89–1.54	0.269
Employed (Farmer)	656	0.85	0.65–1.11	0.91	0.69–1.19	0.475
**Age Group**						
(15, 20)	49	ref		ref		
(20,25)	431	0.78	0.58–1.06	0.75	0.55–1.01	0.057
(25,30)	299	0.80	0.59–1.08	0.75	0.55–1.03	0.072
(30,50)	278	0.66	0.49–0.90	0.64	0.47–0.87	**0.005**
**Anaemia (Hb < 120)**						
No	518	ref		ref		
Yes	539	1.09	0.96–1.23	1.1	0.97–1.25	0.124
**Child sex**						
Female	549	ref		ref		
Male	508	0.96	0.85–1.08	0.95	0.84–1.07	0.418

VIF: ranged from 1.01 to 1.07, Participants were excluded due to missing values in the following variables: anaemia, *n* = 14)

## Discussion

Our study shows that in rural Madagascar, breastfeeding is widely adopted among women, with an encouraging prevalence of continued breastfeeding at 24 months. It also highlights critical issues regarding the delay in the introduction of complementary food. About a quarter of women declared not to have introduced complementary feeding at nine months, three months longer than recommended by international guidelines, with higher frequency among those who were farmers and less educated mothers. The recommendation to introduce complementary food at six months is based on the available literature, which shows a developmental benefit and protection from certain infections at six months compared to earlier periods, but also indicates a negative effect on growth with exclusive breastfeeding beyond six months [22] and additional poor health outcomes, such as anaemia in the child [23]. A nationwide population based cohort study conducted in Korea also found that the introduction of complementary food after seven months was associated with higher frequency of hospitalisation and lower heights, in support of complementary feeding to be started earlier [7]. In a recent review of the literature conducted to develop the WHO guidelines on complementary feeding, the late introduction (after six months) of complementary food was associated to lower length and body mass index but not to stunting, weight or wasting compared to children who were complementary fed before six months. Additionally, they could identify a positive association with episodes of diarrhoea in case of late introduction, which on the other hand we did not find in our study. Finally, they report no association of a delayed introduction of complementary food with several health outcomes, such as anaemia, lower respiratory tract infections or atopic dermatitis. Importantly the researchers highlights the limitations of the findings due mostly to studies not designed to capture the association between the timing of the introduction of complementary feeding and nutritional or health outcomes [6]. This is a similar limitation of our study in which neither the investigation tool nor the initial study design allowed a proper assessment of the health and nutritional consequences of DCF. Despite that, to the best of our knowledge our study is among the few available in Madagascar highlighting important gaps in terms of nutritional goals that should be urgently addressed in the country [2, 14].

In our analysis, farmer mothers reported DCF more frequently, while those with a higher level of education had already introduced complementary food at nine months more frequently. Both educational attainment and employment status are known determinants of breastfeeding practices, with sometimes contrasting results in the different experiences [9]. Better education and employment were positively associated with favourable complementary feeding in some examples such as in Indonesia and Tanzania [24, 25], a trend also seen in HICs [26], which emphasises the role of these individual factors regardless of the welfare of societies. However, clear trends according to type of work, as in the case of the farmers in our study, are not documented for either good practices in complementary feeding or breastfeeding. The WHO guideline panel underscored that the timing of exclusive breastfeeding and complementary feeding have resource implications, especially for women who are workers and those lacking of supporting environment [6]. The vast majority of the Malagasy population (approx. 80%) is employed in agriculture [27], meaning that the association found between being a farmer mother and DCF could have large implication in the country and deserves further investigation. Similarly, the association found between maternal anaemia and DCF. The finding suggests that the pair of maternal anaemia and breastfeeding requires multisectoral interventions that could be based on maternal education, supplementation policies, screening, and monitoring of this clinical condition. This is even more important in the Malagasy context of food insecurity and in view of the high prevalence of anaemia found in our sample, which at 43.6% is among the highest reported globally [28]. It is estimated that only 7.1% of Malagasy women take at least three months of folic acid and iron supplements [17] during pregnancy, while there is no data on the postpartum iron supplementation (in combination or not with folic acid), as recommended by the WHO in the first 6–12 weeks after birth [29]. Improved monitoring and strengthening of these pre- and post-partum nutritional interventions is crucial to reach the nutritional targets set by the WHO and to change the scenario of malnutrition in the country. The year 2025 has been the year set for the evaluation of the six global targets for the improvement of the nutritional status of mothers, infants, and young children. Maternal anaemia, low birth weight, exclusive breastfeeding at six months, stunting in children under five years, paediatric overweight and wasting are the six indicators selected. They are based on a lifecycle approach, which recognises the importance of adequate nutrition starting early in life and the links between the different indicators [2].

Finally, the breastfeeding frequency at 24 months in our cohort is 54.4%, close to the WHO target of 60% by 2030 [12], higher than the 44% global estimate for 2020 and similar to the 56.6% in the Southern and Eastern African region [30] and aligned with the national recent available data. According to the DHS in 2021, 54% of children born in the previous two years were estimated to be exclusively breastfed at six months, with a median breastfeeding duration of 21.9 months and 60.8% continued breastfeeding at the age of two [14]. These estimates are encouraging for the country, but, together with the results on DCF, they also call for some critical reflection on the overall nutritional status of both children and mothers, the biological interactions and areas for public health coordinated interventions.

The positive association between older age and CB is in line with what has been found in other LMICs [31, 32], and offers an opportunity for awareness interventions. Traditionally, older women play an important role as mentors in childcare and breastfeeding practices in Malagasy society [15]. This natural, informal mentoring could be structured into an intergenerational mentoring scheme between older mothers and younger ones. A similar model has been implemented in Zimbabwe for young HIV-positive mothers and has been shown to improve both caregiving practices and maternal disease management [33]. The highest compliance with CB in the 30–49 age group represents an opportunity for the development of the initiatives to support CB and increase awareness which, being embedded in tradition, may be more easily accepted by younger mothers.

The present study, as secondary analysis of a cluster randomised clinical trial, has the strength of having included a big randomised sample in rural Madagascar attending ante-natal care services through highly standardized procedures periodically monitored as part of the data monitoring plan of the trial. At the same time, limitations are not missing and need to be highlighted. As first, since the study was not designed to assess the impact of breastfeeding and complementary feeding practices on child health, it is not possible to draw conclusions on this outcome. In addition, both the data collection instrument and the visit time points were not specifically designed to investigate breastfeeding practices, and there was a lack of items that could have been additionally explored qualitatively or were known from the literature as potential confounders. Data collection in 2020–2021 (corresponding to the T3 visit) was affected by the COVID-19 pandemic, while the high frequency of CB at T3 and the low frequency of exclusive breastfeeding at T4 did not allow multivariate analysis. The generalisability of the findings could be debatable as the study was conducted in three out of 22 regions of the country though the consistency with DHS data confers solidity to them. 

In conclusion our findings show that early feeding practices are to be addressed as a public health priority in Madagascar to improve both women’s and child’s health. Specifically, we believe that two main lines of public health action should be developed and implemented: health education, which could benefit from intergenerational mentoring initiatives; and general improvement and integration of maternal and child health programmes in a lifecycle approach, which could improve the overall nutritional status of women and children.

## Electronic supplementary material

Below is the link to the electronic supplementary material.

Supplementary Material 1

## Data Availability

Research data supporting the findings of this study are available upon reasonable request from the corresponding author.

## Abbreviations

aPR: adjusted Prevalence ratios
BT: Breastfeeding termination
CCA: Circulating Cathodic Antigen
CRCT: cluster randomised clinical trial
CIs: confidence intervals
CB: continued breastfeeding
cPR: Crude Prevalence Ratios
DCF: Delayed complementary feeding
DHS: Demographic and Health Survey
freeBILy: fast and reliable easy-to-use diagnostics for eliminating bilharzia in young children and mothers
GEE: Generalized estimating equations
HR: hazard ratios
Hb: Haemoglobin
HICs: high income countries
KM: Kaplan–Meier
LMICs: low- and middle-income countries
POC: Point of Care
PHCCs: primary health care centres
RDT: Rapid Diagnostic Test
SD: Standard Deviation
WHO: World Health Organization
