# Supplementary material for: Breastfeeding and complementary feeding practices in Madagascar: results of a secondary analysis of the freeBily trial
Source: Int Breastfeed J. 2026 Jun 12;21:57. doi: 10.1186/s13006-026-00861-6 (PMC13270689; doi:10.1186/s13006-026-00861-6)
Supplement: Supplementary file 1 — Supplementary Material 1 [file 13006_2026_861_MOESM1_ESM.docx]

**Breastfeeding practices in Madagascar: results of a secondary analysis of the freeBILy trial**

Valentina Marchese^1,2^, André Brito^1,2^, Raphäel Rakotozandrindrainy^3^, Norbert Georg Schwarz^4^, Njary Rakotozandrindrainy^3^, Tahinamandranto Rasamoelina^5^, Mala Rakoto Andrianarivelo^5^, Jeannine Solonirina^3^, Elveric Fesia Ratiaharison^6^, Mickael Radomanana^3^, Tiana Randrianarisoa^6^, Philipp Klein ^1,2^, Jule Hameister^1^, Leonard Gunga^1^, Irina Kislaya ^4,2^, Anna Jaeger^4,2^, Govert J van Dam^7^, Jürgen May^4,2^, Rivo Andry Rakotoarivelo^6^, the freeBILy consortium, Daniela Fusco^1,2^

**Corresponding author:**

Daniela Fusco

[Fusco@bnitm.de](mailto:Fusco@bnitm.de)

**Table 1S: Prevalence of continued (CB) and Exclusive (EB) breastfeeding stratified by maternal and child characteristics at T3**

| Characteristics | Breastfeeding (CB) | | | | Exclusive breastfeeding (EB) | | | |
| --- | --- | --- | --- | --- | --- | --- | --- | --- |
|  | **n** | **Prevalence** | **CI95%** | | **n** | **Prevalence** | **CI95%** | |
| Urbanization |  |  |  |  |  |  |  |  |
| Rural | 1380 | 98.7 | 97.5 | 99.4 | 389 | 28.3 | 11.5 | 54.5 |
| Urban | 468 | 99.2 | 97.6 | 99.7 | 90 | 19.2 | 4.7 | 53.6 |
| Education level |  |  |  |  |  |  |  |  |
| Never went to school | 66 | 98.5 | 89.4 | 99.8 | 25 | 37.9 | 14.5 | 68.7 |
| Primary School | 894 | 98.0 | 96.5 | 98.9 | 264 | 29.6 | 13.3 | 53.5 |
| Secondary school or higher | 888 | 99.7 | 99.0 | 99.9 | 190 | 21.4 | 9.9 | 40.4 |
| Employment |  |  |  |  |  |  |  |  |
| Not employed | 117 | 98.3 | 96.8 | 99.1 | 11 | 21.4 | 9.9 | 40.4 |
| Employed (Non-farmer) | 389 | 100.0 | na | na | 45 | 29.6 | 13.3 | 53.5 |
| Employed (Farmer) | 1319 | 98.5 | 97.2 | 99.2 | 419 | 37.9 | 14.5 | 68.7 |
| Age Group |  |  |  |  |  |  |  |  |
| [15,20] | 253 | 98.4 | 95.0 | 99.5 | 78 | 31.0 | 14.8 | 53.6 |
| (20,25] | 636 | 99.8 | 98.9 | 100.0 | 160 | 25.2 | 11.4 | 47.1 |
| (25,30] | 472 | 98.1 | 95.9 | 99.2 | 121 | 25.7 | 11.7 | 47.5 |
| (30,50] | 487 | 98.4 | 96.2 | 99.3 | 120 | 24.6 | 10.7 | 47.3 |
| Anaemia (Hb<120 g/L) |  |  |  |  |  |  |  |  |
| No | 923 | 98.4 | 97.1 | 99.1 | 285 | 31.0 | 14.6 | 54.0 |
| Yes | 797 | 99.1 | 97.9 | 99.7 | 192 | 24.1 | 10.6 | 46.1 |
| Child sex |  |  |  |  |  |  |  |  |
| Female | 902 | 98.7 | 97.2 | 99.4 | 237 | 26.3 | 12.4 | 47.5 |
| Male | 946 | 99.0 | 97.9 | 99.5 | 242 | 25.7 | 11.5 | 47.9 |
| Overall | 1848 | 98.8 | 97.9 | 99.4 | 479 | 25.7 | 11.8 | 47.1 |

#Estimates based on n<10 should be interpreted with caution

**Table 2S: Prevalence of continued (CB) and exclusive (EB) breastfeeding stratified by maternal and child characteristics at T4**

| Characteristics | Breastfeeding (CB) | | | | Exclusive breastfeeding (EB) | | | |
| --- | --- | --- | --- | --- | --- | --- | --- | --- |
|  | n | Prevalence | CI95% | | n | Prevalence | CI95% | |
| Urbanization |  |  |  |  |  |  |  |  |
| Rural | 936 | 57.7 | 46.8 | 68.0 | 46 | 2.8 | 0.8 | 9.2 |
| Urban | 333 | 46.7 | 29.3 | 65.0 | 32 | 4.5 | 0.6 | 28.7 |
| Education level |  |  |  |  |  |  |  |  |
| Never went to school | 56 | 62.9 | 46.3 | 76.9 | 5 | 5.7 | 1.6 | 18.1 |
| Primary School | 651 | 57.5 | 45.8 | 68.4 | 50 | 4.4 | 1.6 | 11.7 |
| Secondary school or higher | 562 | 50.5 | 42.0 | 59.0 | 23 | 2.1 | 0.5 | 8.4 |
| Employment |  |  |  |  |  |  |  |  |
|  | 98 | 43.8 | 37.4 | 50.3 | 3 | 1.3 | 0.2 | 8.0 |
| Employed (Non-farmer) | 237 | 46.1 | 37.0 | 55.5 | 13 | 2.5 | 0.4 | 15.4 |
| Employed (Farmer) | 934 | 58.5 | 46.3 | 69.8 | 62 | 3.9 | 1.4 | 10.5 |
| Age Group |  |  |  |  |  |  |  |  |
| [15,20] | 34 | 41.0 | 30.4 | 52.5 | 2 | 2.4 | 0.3 | 16.3 |
| (20,25] | 450 | 51.0 | 40.4 | 61.5 | 27 | 3.1 | 1.0 | 9.2 |
| (25,30] | 350 | 53.8 | 44.4 | 62.9 | 26 | 4.0 | 1.2 | 12.5 |
| (30,50] | 435 | 60.6 | 49.3 | 70.8 | 23 | 3.2 | 1.2 | 8.5 |
| Anaemia (Hb<120 g/L ) |  |  |  |  |  |  |  |  |
| No | 670 | 56.4 | 47.2 | 65.2 | 47 | 4.0 | 1.2 | 12.7 |
| Yes | 593 | 52.4 | 41.0 | 63.6 | 31 | 2.7 | 1.0 | 7.5 |
| Child sex |  |  |  |  |  |  |  |  |
| Female | 620 | 52.7 | 42.8 | 62.5 | 37 | 3.2 | 1.1 | 8.8 |
| Male | 649 | 56.0 | 45.8 | 65.8 | 41 | 3.6 | 1.1 | 10.7 |
| Overall | 1269 | 54.4 | 44.5 | 63.9 | 78 | 3.4 | 1.1 | 9.7 |

#Estimates based on n<10 should be interpreted with caution

**Table S3. Cumulative breastfeeding probability by time since delivery (in months) estimated using the Kaplan–Meier (KM) method**

| Time of Breastfeeding (months) | Breastfeeding | Breastfeeding termination | Probability | 95% CI |  |
| --- | --- | --- | --- | --- | --- |
| 2 | 2334 | 1 | 1.000 | 0.999 | 1.000 |
| 3 | 2333 | 1 | 0.999 | 0.998 | 1.000 |
| 9 | 2332 | 2 | 0.998 | 0.997 | 1.000 |
| 10 | 2330 | 1 | 0.998 | 0.996 | 1.000 |
| 11 | 2329 | 1 | 0.997 | 0.995 | 0.999 |
| 12 | 2328 | 7 | 0.994 | 0.991 | 0.997 |
| 13 | 2321 | 2 | 0.994 | 0.990 | 0.997 |
| 14 | 2319 | 9 | 0.990 | 0.986 | 0.994 |
| 15 | 2310 | 13 | 0.984 | 0.979 | 0.989 |
| 16 | 2297 | 10 | 0.980 | 0.974 | 0.986 |
| 17 | 2287 | 14 | 0.974 | 0.967 | 0.980 |
| 18 | 2273 | 137 | 0.915 | 0.904 | 0.927 |
| 19 | 2136 | 59 | 0.890 | 0.877 | 0.903 |
| 20 | 2077 | 173 | 0.816 | 0.800 | 0.832 |
| 21 | 1904 | 60 | 0.790 | 0.774 | 0.807 |
| 22 | 1844 | 106 | 0.745 | 0.727 | 0.763 |
| 23 | 1738 | 165 | 0.674 | 0.655 | 0.693 |
| 24 | 1515 | 260 | 0.558 | 0.538 | 0.579 |
| 25 | 511 | 44 | 0.510 | 0.488 | 0.534 |
